# Pericardial Metastasis Induced Tamponade from Urothelial Carcinoma: A Rare Entity

**DOI:** 10.1155/2016/6162732

**Published:** 2016-04-11

**Authors:** Rafay Khan, Waqas Jehangir, Sunil Tulpule, Mohamed Osman, Shilpi Singh, Shuvendu Sen

**Affiliations:** Department of Internal Medicine, Raritan Bay Medical Center, 530 New Brunswick Avenue, Perth Amboy, NJ 07733, USA

## Abstract

Urothelial carcinoma in a few cases may result in cardiac metastasis. A rare presentation of this condition is its diagnosis as a result of cardiac tamponade. Tamponade is an unusual entity as a result of urothelial carcinoma and has only been reported in four cases. There have also been only a total of fifteen cases of cardiac metastasis from this form of malignancy. It is through this discussion that we emphasize the importance of early detection and monitoring of cardiac symptoms with the implementation of echocardiogram imaging. Although not feasible in all patients it may be considered in those presenting with cardiac and pulmonary symptoms. In this case we discuss the presentation of a 71-year-old gentleman with a history of urothelial carcinoma after cystectomy and while on chemotherapy presented with new onset atrial fibrillation and later was diagnosed with cardiac tamponade as a result of malignant metastasis.

## 1. Introduction

In most cases of urothelial carcinoma, cardiac metastasis goes unnoticed and can be an incidental finding upon autopsy. It has been shown that ten percent of autopsied cases of urothelial carcinoma have revealed cardiac metastases [1, 2]. After a review of the literature and to the best of our knowledge, there have only been four cases of cardiac tamponade secondary to urothelial carcinoma and this is only the second case diagnosed with cardiac metastasis while the patient was still alive. Through this discussion we will illustrate the importance of keeping cardiac issues as a result of metastasis as part of the differential diagnosis in any patient with urothelial carcinoma.

## 2. Case Presentation

A 71-year-old male with past medical history significant for bladder cancer status after radical cystectomy on chemotherapy, diabetes, coronary artery disease, and hypertension presented to the emergency room with complaints of fever and shivering. Three days prior to admission, his oncologist diagnosed him with anemia. He had complaints of cough with white sputum consisting of up to half a cup per day. He denied any shortness of breath, diarrhea, hematuria and dysuria, or weight loss.

**Figure 1:**
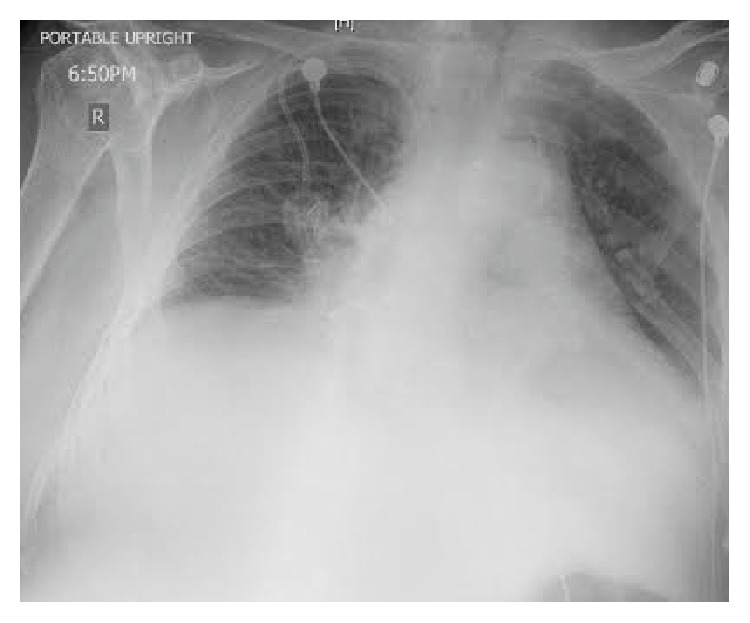
Chest X-ray suggestive of an enlarged cardiac silhouette.

Upon physical examination patient was febrile with a temperature of 100.7 F, blood pressure of 110/62 mmHg, heart rate of 114/min, and respiratory rate of 20/min and had O_2_ saturation of 100% on two liters' nasal cannula. Pertinent findings revealed irregular rate and rhythm with distant heart sounds. The remainder of the physical exam was otherwise unremarkable. Laboratory data demonstrated hemoglobin of 7.0 g/dL, hematocrit of 21.7%, white blood cell count of 5.7 K/*μ*L with absolute neutrophil count 5.0 K/*μ*L, and platelet count 37 K/*μ*L. Basic metabolic panel revealed glucose of 100 mg/dL, blood urea nitrogen of 27 mg/dL, creatinine of 0.7 mg/dL, calcium 8.0 mg/dL, albumin 2.9 g/dL, sodium 134 mmol/L, potassium 4.4 mmol/L, chloride 103 mmol/L, and bicarbonate 20 mmol/L. Electrocardiogram showed atrial fibrillation with low voltage. Chest X-ray showed marked enlargement of the cardiac silhouette with a possibility of large pericardial effusion (Figure 1).

**Figure 2:**
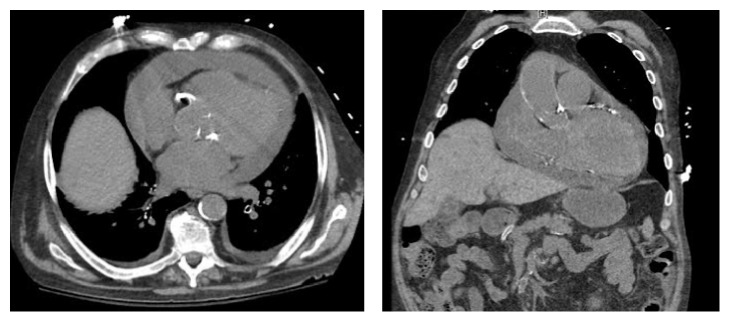
CT imaging demonstrated large pericardial effusion.

The patient was admitted to the intensive care unit for sepsis and large pericardial effusion. He was started on empiric antibiotics. Computed tomography scan of the chest was ordered to rule out any metastasis which revealed a large pericardial effusion that was 2.2-cm in thickness (Figure 2). Echocardiography demonstrated large circumferential pericardial effusion.

**Figure 3:**
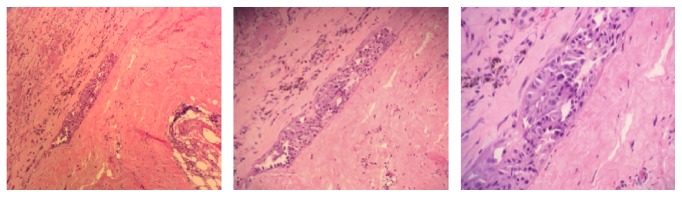
Pericardial biopsy illustrating pathology positive for metastatic urothelial carcinoma.

A cardiothoracic surgeon was called on consult and the patient underwent a pericardial window where 800 mL of hemorrhagic pericardial effusion was drained. Pericardial biopsy illustrated metastatic urothelial carcinoma (Figure 3). Immunostains were performed which were positive for pancytokeratin, CK7, CK20, and uroplakin and negative for PSA. Patient remained critical and, despite all the efforts, the patient deteriorated and expired over the course of a few days due to his relatively poor prognosis.

## 3. Discussion

Bladder cancer is the fourth most common cancer in men; however metastasis to the heart is typically an incidental finding and is a rare occurrence. Previous cases such as that from Lin and Telen [3] have reported metastasis into the right ventricle, which was similarly seen in a total of fourteen cases which have been reported in the literature, seven of which began from the renal pelvis and seven began from the bladder. To the best of our knowledge this is only the second case reporting cardiac tamponade as a result of cardiac metastasis that was diagnosed prior to the patient's death and autopsy.

Pericardial effusion was found in 25.4% of autopsied cases demonstrating cardiac involvement; however cases presenting as cardiac tamponade were rare [1]. The three cases which demonstrated cardiac tamponade included Fabozzi et al. [4] which illustrated anaplastic cells in a pericardial window patient with 700 cc of hemorrhagic fluid removed; unfortunately the patient passed six months later. Islam and Ahmedani [5] described the case of a 39-year-old male with left ventricular metastasis stemming from a primary site located in the renal pelvis where 200 cc was removed via pericardiocentesis; however in this case the cytology was negative. The third case by Spiliotopoulos et al. [6] discussed the presentation of a 66-year-old male with right atrial metastasis from a bladder primary draining 2000 mL of blood fluid who underwent chemotherapy and survived for over a year after. Thus, this is only the fourth reported case of this form of malignancy and only the third stemming from specifically the bladder resulting in cardiac tamponade.

The management of TCC with cardiac metastasis remains unclear at this time. The majority of patients diagnosed with its spread to the heart have died within few weeks after diagnosis. Chemotherapy as well as resection has been attempted in a few cases without significant improvement in the patients condition. One patient however did remain asymptomatic for one year with up to a forty percent reduction in the cardiac tumor size before deterioration after the use of gemcitabine-carboplatin based chemotherapy and another demonstrated some improvement for seven months prior to deterioration [6, 7]. In patients with the diagnosis of urothelial carcinoma, whether there are local or distant signs of metastasis or even cardiac symptoms it may be vital to consider early echocardiography as it may prevent long term complications. However, cardiac metastasis itself remains a rare clinical finding although it is found in ten percent of patients with urothelial carcinoma upon autopsy.

## 4. Conclusion

Through this case presentation, it should remain a concern in a physician's management to keep cardiac metastasis in mind as a patient's symptoms may not be very typical of tamponade and may not always include signs of dyspnea and shortness of breath. Early echocardiography may be considered in patients with a history of this malignancy with any signs of cardiac or pulmonary complications or symptoms. The prognosis of cardiac metastasis from urothelial carcinoma is very poor and thus treatment modalities including pericardiocentesis, pericardial window, and chemotherapy must be properly addressed and chosen carefully.
